# Transcriptomics and Identification of the Chemoreceptor Superfamily of the Pupal Parasitoid of the Oriental Fruit Fly, *Spalangia endius* Walker (Hymenoptera: Pteromalidae)

**DOI:** 10.1371/journal.pone.0087800

**Published:** 2014-02-05

**Authors:** Yuping Zhang, Yuan Zheng, Dunsong Li, Yilin Fan

**Affiliations:** 1 Plant Protection Institute, Guangdong Academy of Agricultural Sciences, Guangzhou, China; 2 Guangdong Provincial Key Laboratory of High Technology for Plant Protection, Guangzhou, Guangdong, China; 3 Guangdong Academy of Agricultural Sciences, Guangzhou, China; Virginia Tech, United States of America

## Abstract

**Background:**

The oriental fruit fly, *Bactrocera dorsalis* Hendel, causes serious losses to fruit production and is one of the most economically important pests in many countries, including China, *Spalangia endius* Walker is a pupal parasitoid of various dipteran hosts, and may be considered a potentially important ectoparasitic pupal parasitoid of *B. dorsalis*. However, lack of genetic information on this organism is an obstacle to understanding the mechanisms behind its interaction with this host. Analysis of the *S. endius* transcriptome is essential to extend the resources of genetic information on this species and, to support studies on *S. endius* on the host *B. dorsalis*.

**Methodology/Principal Findings:**

We performed *de novo* assembly RNA-seq of *S. endius*. We obtained nearly 10 Gbp of data using a HiSeq platform, and 36319 high-quality transcripts using Trinity software. A total of 22443 (61.79%) unigenes were aligned to homologous sequences in the jewel wasp and honeybee (*Apis florae*) protein set from public databases. A total of 10037 protein domains were identified in 7892 *S. endius* transcripts using HMMER3 software. We identified expression of six gustatory receptor and 21 odorant receptor genes in the sample, with only one gene having a high expression level in each family. The other genes had a low expression level, including two genes regulated by splicing. This result may be due to the wasps being kept under laboratory conditions. Additionally, a total of 3727 SSR markers were predicted, which could facilitate the identification of polymorphisms and functional genes within wasp populations.

**Conclusion/Significance:**

This transcriptome greatly improves our genetic understanding of *S. endius* and provides a large number of gene sequences for further study.

## Introduction

Biological control of insect pests is one of the most cost-effective and environmentally sound methods of pest management. However, several studies have reported that the differences in the habitat of the host can induce differentiation in the parasitoid resulting in the development of different genotypes in the wasp population [1]–[3]. Exotic species may not be an effective method of controlling local insect pests, as the efficacy of biological control of parasitoid insects relies to a great extent on their adaptability to the host. Therefore, the localization of the parasitoid insect is the most important factor for controlling the insect pests.

The oriental fruit fly, *Bactrocera dorsalis* Hendel, is one of the most important quarantine pests in Asian countries. It can infest on up to 250 different types of fruits and vegetables, causing severe economic loss [4]. In recent years, damage by oriental fruit flies caused tens of billions of direct economic loss in China. In most areas in southern China, populations of the oriental fruit fly have evolved varying levels of resistance to organophosphorus, pyrethroids and abamectin pesticides [5]–[12]. Male lures as an attractant are mainly used for controlling this insect, but the effects of these are not ideal [13], [14]. Bagging is a better prevention measure, but owing to its heavy workload and high cost, it is generally applied only for fruits of larger size and greater economic value [15]. Therefore, it is important to formulate simple and effective strategies for agricultural pest control.

In many countries, for example Hawaii, Vietnam and Thailand, the use of parasitoids to control *B. dorsalis* has achieved remarkable results, mostly by using the larval parasitoid [16]. Resources of the pupal parasitoid are very scarce. *Spalangia endius* Walker is a pupal parasitoid of various dipteran insects [17]–[21], including tephritid fruit flies [4]. In Thailand, *S. endius* has been found in mixed infestations of the tephritid fruit flies *B. correcta* and *B. dorsalis*, and may be considered a potential biological control agent [22]. We successfully located *S. endius* in South China and reared it under laboratory conditions, suggesting that. *S. endius* could be an ideal parasitoid wasp for biological control of the oriental fruit fly in China. However, there have been no reports of local parasitoid wasps successfully controlling oriental fruit fly populations, or how this wasp may adapt to the development of new host resources, emigration, differentiation and habitat. Compared with the model insect species whose genomes have been sequenced, such as *Drosophila melanogaster*, *Anopheles gambiae*, *Bombyx mori* and *Nasonia vitripennis*, the genomic sequence resources for *S. endius* are limited.

In this study, we used *de novo* transcriptome analysis to obtain basic data. The resulting annotated transcriptome sequences extend the genomic resources available for researchers studying the parasitoid *S. endius* of *B. dorsalis*, and may provide a rapid approach for future studies into the molecular mechanisms of its adaption to different hosts.

## Materials and Methods

### Ethics Statement

No specific permits were required for the described field studies. No specific permissions were required for these insects.

### Flies and Parasitoids

The oriental fruit fly, *B. dorsalis*, was reared in the laboratory on an artificial diet, and originated from a population collected at Guangzhou, China in April 2009 that were reared on a banana and maize-based artificial diet. About 600 flies were housed in a screen cage (50×50×30 cm^3^) and supplied with water, sugar and yeast extract. The adult insects were reared in laboratory conditions controlled at 25–27°C under a photoperiod of 14 h light - 10 h dark (14L:10D) and 60–80% relative humidity (RH). A small plastic container (4 cm diameter×4 cm), containing moistened coarse sand, was used as the oviposition substrate. Two to three pieces of banana were used to stimulate oviposition. Fresh eggs were incubated on an artificial diet in plastic containers (20×12×4 cm) and reared in containers under laboratory conditions. When the larvae started to pupate, each rearing container was placed into a fiberglass box (45×25×15 cm^3^) containing 2–3 cm of sand, so that fly puparia could be easily collected. The fly puparia were collected daily and put into a petri dish (5 cm diameter) containing moistened filter paper. The petri dish was placed in an incubator controlled under laboratory conditions.

A laboratory population of *S. endius* Walker was primarily obtained from oriental fruit fly infesting guava growing in Guangzhou. The *S. endius* colony was maintained on pupae of the oriental fruit fly, *B. dorsalis*, which were reared on bananas under laboratory conditions at 26±2°C and 70±10% RH. The *S. endius* colony was reared on *B. dorsalis* by exposing about 100–200 two- to three-day-old *B. dorsalis* puparia to about 100 pairs of zero- to ten-day-old adult wasps of *S. endius* on a petri dish (9 cm diameter) with water and honey provided. All rearing and experiments were conducted under the same laboratory conditions described above. The lifecycle of the wasps was 18–20 days under 20–25°C, 80–90% RH and 16 h photophase[23]. Adult wasps of *S. endius* were collected between emergence and one day of age. The wasps were frozen immediately in liquid nitrogen, and stored at −80°C for future RNA extraction.

### RNA Extraction, RNA-Seq Library Preparation and Sequencing

The RNA extraction, cDNA library preparation and sequencing are described briefly as fellows. Firstly, total RNA was extracted from prepared samples using TRIzol reagents (Invitrogen, USA) according to the manufacturer’s instructions. Each RNA sample was subjected to DNase digestion (Takara, Dalian, China) to remove any remaining DNA. Secondly, at least 1 µg of total RNA was used to prepare a cDNA library using TruSeq RNA library preparation kits v2 (Low-Throughput protocol; Illumina) following the manufacturer’s instructions. Next, the quality and quantity of the library were estimated on an Agilent Bioanalyzer using high sensitivity DNA chips. Finally, each fragment from a qualified library underwent pair-end sequencing (PE) via the Illumina HiSeq™ 2000 at the Beijing Genomics Institute (Shenzhen, China).

### RNA-Seq Data Filter

To ensure the accuracy of subsequent analysis, raw sequences were cleaned to remove adaptors and sequencing errors. Reads were removed that contained the sequencing adaptor, more than 5% unknown nucleotide and more than 20% bases of low quality (quality scores in Phred scale less than 10). This output was called ‘clean reads’, which was used for the following analysis. All the reads were deposited in the National Center for Biotechnology Information (NCBI) and can be accessed in the Short Read Archive (SRA) under accession number: SRR1038395.

### Transcript Assembly

The publicly available program Trinity (trinityrnaseq_r2012-05-18; http://trinityrnaseq.sourceforge.net/) was used for *de novo* assembly of clean reads to generate a set of transcripts [24]. The following parameters were used in Trinity: min_glue = 3, V = 10, edge-thr = 0.05, min_kmer_cov = 3, path_reinforcement_distance = 85, group_pairs_distance = 250, and the other parameters were set as the default. Next, any redundant fragments were removed javascript:void (0);by TGICL (TGI Clustering tools) and Phrap assembler [25]. The following parameters were used to ensure a high quality of assembly: a minimum of 95% identity, a minimum of 35 overlapping bases, a minimum of 35 scores and a maximum of 25 unmatched overhanging bases at sequence ends. Finally, based on sequence similarity, the transcripts were divided into two classes: cluster (prefixed with ‘CL’) and singleton (prefixed with ‘unigene’). In a cluster, the similarity between transcripts was more than 70%.

To evaluate the accuracy of the assembled transcripts, the method of Liu and colleagues [26] was used. All the usable sequencing reads were realigned onto the transcripts using SOAPaligner (Release 2.21, 02-14-2011), allowing up to three base mismatches and a minimum length of 40 bp. The read coverage of each transcript was calculated, excluding the 40 bp at both ends of the transcript. If the transcript was completely covered by at least one read, this transcript was defined as positive.

### Annotation, Predicted CDS and Gene Expression

The transcripts were annotated using public animal databases and whole protein sets of related species. Firstly, the transcripts were aligned to three public protein databases, NCBI non-redundant (Nr) database, Swiss-Prot protein database (Swiss-Prot), and Kyoto Encyclopedia of Genes and Genomes (KEGG) and two whole protein sets of related species (WPR; *N. vitripennis* and *A. mellifera*) by blastx, and the cut-off E-value was 1×10^−5^. The best hit was used to determine the sequence direction and CDS (Coding sequences) of transcripts, and the peptide sequences were translated using standard codons. When different databases conflicted, the results were prioritized in the following order: WPR, nr, Swiss-Prot and KEGG. When a transcript was not covered in blastx, it was predicted by ESTScan. The shortest CDS were at least 60 bp. Based on Nr annotation, GO annotation was analyzed by Blast2GO software (v2.5.0). In addition, transcripts were annotated with the NCBI non-redundant nucleotide (Nt) database using blastn. We annotated the motifs and domains using Pfam 27.0 with a cut-off of 1×10^−3^ [27].

Gene expression levels were calculated by FPKM (fragment per kilobase per million mapped fragments) [28], [29]. The clean reads were mapped to all transcripts using the SOAPaligner (Release 2.21, 08-13-2009), allowing mismatches of no more than three bases. For gene expression analysis, the number of uniquely matched reads was calculated and then normalized to give the FPKM.

### Gene Family Comparative and *S. endius*-specific Genes

To identify the *S. endius*-specific gene families, we selected the following reference species to represent sequenced related species and model species: *N. vitripennis* (Hymenoptera: Pteromalidae), *A. mellifera* (Hymenoptera: Apidae) and *D. melanogaster* (Diptera: Drosophilidae). For comparative analysis, we used the following pipeline to cluster individual genes into gene families using Treefam [30]: 1) we collected protein sequences longer than 33 amino acids from these four species, with the longest protein isoform being retained for each gene; 2) blastp was used to align all protein sequences against a database containing a protein dataset of all species with an e-value of 1×10^−7^, combined with fragmental alignments for each gene pair by Solar [31]; 3) gene families were extracted by hcluster with default parameters.

### Identification of the Chemoreceptor Superfamily Transcripts

To identify the chemoreceptor superfamily, all transcripts were realigned onto the orthologous protein sets of *N. vitripennis* and *A. mellifera* identified by Robertson and colleagues [32], using blastx with a cut-off of 1×10^−10^. Putative alternative splice variants were filtered based on sequence similarity, using the criteria of an overlap ratio no less than 70% to any sequences and an identity no less than 0.95. The longest protein isoform was retained for each gene. The phylogeny tree was reconstructed from all the genes, which contains all the identified chemoreceptor superfamily genes in *S. endius*, *N. vitripennis* and *A. mellifera*, using PhyNJ with default parameters [33].

### cDNA- Simple Sequence Repeat (cSSR) Discovery

cSSRs were identified with a Perl script of MIcroSAtellite (MISA), using unigenes for reference. Mono-, di-, tri-, tetra-, penta- and hexa-nucleotide sequences, with a minimum repeat number of 12, 6, 5, 5, 4 and 4, respectively, were applied as the search criteria (http://pgrc.ipk-gatersleben.de/misa/). Primer3-2.3.4 was used to design PCR primers with default settings. Primers were filtered based on the following criteria: (1) no SSRs in the primer; (2) three mismatches at the 5′ -site and one mismatch at the 3′ -site were allowed when aligning primers to unigenes; (3) each primer could only map to one unigene [34].

## Results

### RNA-Seq Data Filtering and Assembly

**Figure 1:**
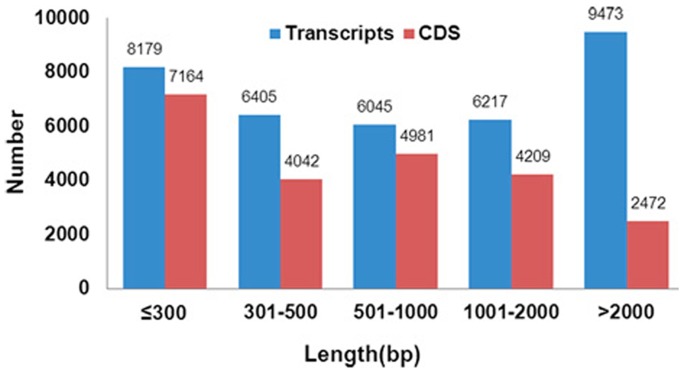
Size distribution of the transcripts and CDSs. The blue and red bars indicate unigenes and CDS, respectively.

Using Illumina sequencing, each fragment from an approximately 100–300 bp insert library was used in paired-end sequencing (PE), and the length of each sequence read was 90 bp. After data filtering, we generated 55.5 million clean reads, a total of 9983 million bases with more than 97% Q20 bases (base quality more than 20 and an error rate of less than 0.01), and these data were used for *de novo* assembly (Table 1) by Trinity [24]. A total of 36319 transcripts of more than 200 bp in length were obtained after removal of redundant reads, including 3266 clusters (containing 10168 transcripts) and 26151 singletons. The total length and N50 length (N50 size of transcripts was the length such that 50% of the assembled genome lies in blocks of the N50 size or longer) were 54072690 bp and 2956 bp, respectively (Figure 1 and Table 1). A total of 93.15% of the reads were realigned back to unigenes using SOAP2, and this suggests that we obtained a majority of transcripts in the present data. The accuracy of assembly evaluation showed that 98.91% of transcripts were completely covered by at least one read (Table 1).

**Table 1:** Summary of sequencing and assembly for *S. endius.*

Reads	
Length (bp)	90+90
Total Number	55462088
Total Bases (bp)	9983175840
GC percentage	40.14%
Q20 percentage	97.16%
Transcripts (≥200 bp)	
Total Number	36319
Total Length (bp)	54072690
Clusters	3266
Singletons	26151
N50 Length (bp)	2956
Total Used Reads	93.15%
Accuracy of assembly	98.91%

### Annotation and CDS Prediction

For annotation, homologs of the unigenes were searched for in Nr, Swiss-Prot, KEGG, COG and Nt using BLAST with an E-value threshold of 1×10^−5^. A total of 22294 (61.38%) unigenes were annotated to at least one of the five databases (Table 2 and Table S1). Among them, 20901 transcripts could be annotated to the Nr database, in which 68.90% of transcripts showed a best hit belonging to *N. vitripennis*, just an additional 1.70% showed one that belonged to *A. florea*. Both of these insects are Hymenoptera with sequenced genomes. This implies that *S. endius* is more closely related at the genetic level to the jewel wasp *N. vitripennis* than the honeybee *A. florea*.

**Table 2:** Summary of annotations of *S. endius* transcripts.

Annotated databases	Sequences
NT	16160
NR	20901
UniProt/Swiss-Prot	17086
KEGG	15570
COG	10157
GO	9443
*N. vitripennis*	14862
*A. mellifera*	10641
Total	22443

To identify homologous genes in the two related species, the transcripts were realigned to the whole proteome of *N. vitripennis* and *A. florea*. 14862 unigenes realigned to the jewel wasp genome and 10641 unigenes realigned to the honeybee genome using blastx (Table 2). A total of 22443 (61.79%) unigenes were aligned to homologous sequences in the public databases of the jewel wasp protein set and the honeybee protein set (Table 2). The remaining unigenes (38.21%) may be novel transcripts and genes specific to *S. endius*.

A total of 22868 unigenes were predicted to have CDS (Coding sequences) no less than 100 bp, using blastx and ESTscan (Figure 1). 21 063 unigenes with homologous matches in the four protein databases were identified to be CDS. Other unigenes were processed with ESTScan and a total of 1 805 unigenes were detected. The unigenes without identified coding regions were likely to be too short to meet the criteria for CDS prediction, or may be non-coding RNAs. These putative non-coding RNAs need to be validated in a future study.

### Protein Domains

A total of 10037 protein domains were identified in 7892 *S. endius* transcripts using HMMER3 software (Table 3 and Table S2). Among these domains, a total of 182 WD40 proteins were identified (Table 3). The underlying common function of all WD40-repeat proteins is the coordination of multi-protein complex assemblies, where the repeating units serve as a rigid scaffold for protein interactions. The specificity of the proteins is determined by the sequences outside the repeats themselves. Examples of such complexes are G proteins (where the beta subunit is a beta-propeller), TAFII transcription factor, and E3 ubiquitin ligase [35], [36].

**Table 3:** Summary of top 15 domains predicted in *S. endius* transcripts.

Domain accession	Domain name	Domain description	Occurrence	Common function
PF00400.27	WD40	WD domain, G-beta repeat	182	Co-ordinating multi-protein complex assemblies; the repeating units serve as a rigid scaffold for protein interactions
PF00069.20	Pkinase	Protein kinase domain	130	Phosphorylation; activate or de-activate an enzyme
PF00076.17	RRM_1	RNA recognition motif	125	Involved in pre-mRNA processing and transport, regulation of stability and translational control
PF13465.1	zf-H2C2_2	zinc finger protein Superfamily	98	Binding target sites
PF00067.17	p450	Cytochrome P450	98	Metabolism of xenobiotics
PF12796.2	Ank_2	Ankyrin repeats	91	Protein-protein interaction platforms
PF00096.21	zf-C2H2	zinc finger protein Superfamily	90	Binding target sites
PF00328.17	His_Phos_2	Histidine phosphatase superfamily	75	Dephosphorylation; activate or de-activate an enzyme
PF00078.22	RVT_1	RNA-dependent DNA polymerase	73	Utilize reverse transcriptase to move from one position to another via an RNA intermediate in the genome
PF00083.19	Sugar_tr	Sugar (and other) transporter	67	Transport of nutrients
PF00089.21	Trypsin	Trypsin	65	Hydrolyses proteins; immune defense in insects
PF07004.7	SHIPPO-rpt	Sperm-tail PG-rich repeat	65	Unknown function
PF00071.17	Ras	Ras family	61	Involved in insect development
PF00018.23	SH3_1	SH3 domain	55	Regulatory proteins of signaling pathways
PF00271.26	Helicase_C	Helicase conserved C-terminal domain	55	Helicase conserved C-terminal domain

Protein kinase (130) and proteins of the Histidine phosphatase superfamily (75) are involved in signal transduction pathways, development, cell division and metabolism in higher organisms [37], [38]. Protein kinases are a group of enzymes that add a phosphate group to proteins in a process called phosphorylation, while the actions of histidine phosphatase superfamily members are directly opposite to that of phosphorylases and kinases. The addition of a phosphate group may activate or de-activate an enzyme, for example in kinase signaling pathways [39], or enable a protein-protein interaction to occur, as in SH2 domains [40].

In total, 125 RNA recognition motifs (RRMs) were predicted in this study. These proteins are involved in pre-mRNA processing and transport, regulation of stability, and translational control [41], [42]. RRMs are reported to be involved in male courtship and vision in *D. melanogaster* [43], [44]. Mutations in RRMs of *D. melanogaster* resulted in reduced viability and female sterility, with abnormal wing and mechanosensory bristle morphology [41].

The C2H2-zf clan of zinc finger proteins had the highest result, with seven family members identified in the present study: zf-H2C2_2 (98), zf-H2C2_5 (21), zf-H2C2 (6), zf-C2H2 (90), zf-C2H2_4 (18), zf-C2H2_jaz (14), zf-C2H2_6 (10). The vast majority of these typically function as interaction modules, binding DNA, RNA, proteins, or other small useful molecules. Variations in structure primarily serve to alter the binding specificity of a particular protein [45].

The cytochrome P450 domains (98) were predicted in the derived transcriptomic sequences of *S. endius*. Insect cytochrome P450s are reported to be involved in the metabolism of xenobiotics, and induced levels are correlated with pesticide resistance and plant allelochemicals [46], [47]. For example, CYP6G1 is linked to insecticide resistance in DDT-resistant *D. melanogaster* [48], and CYP6Z1 in the mosquito malaria vector *Anopheles gambiae* is capable of directly metabolizing DDT [49].

Ankyrin repeats (91) typically fold together to form a single, linear solenoid structure called ankyrin repeat domains. These domains are one of the most common protein-protein interaction platforms in nature. Some evidence suggests that the C-terminus forms the folding nucleation site, based on synthesis of truncated versions of natural repeat proteins [50] and on the examination of phi values (which is an experimental protein engineering method used to study the structure of the folding transition state in small protein domains that fold in a two-state manner) [51].

Trypsin (65) was identified in *S. endius* sequences, which is known to be involved in regulation of immune and developmental processes in the diapausing pupae of the Onion maggot and *Pseudaletia separata* [52], [53].

SH3 domains (55) are found in proteins of signaling pathways that regulate the cytoskeleton, Ras protein, Src kinase, and many others. They also regulate the activity state of adaptor proteins and other tyrosine kinases, and are thought to increase the substrate specificity of some tyrosine kinases by binding far away from the active site of the kinase [54].

### Gene Family Comparative and *S. endius*-specific Genes

**Figure 2:**
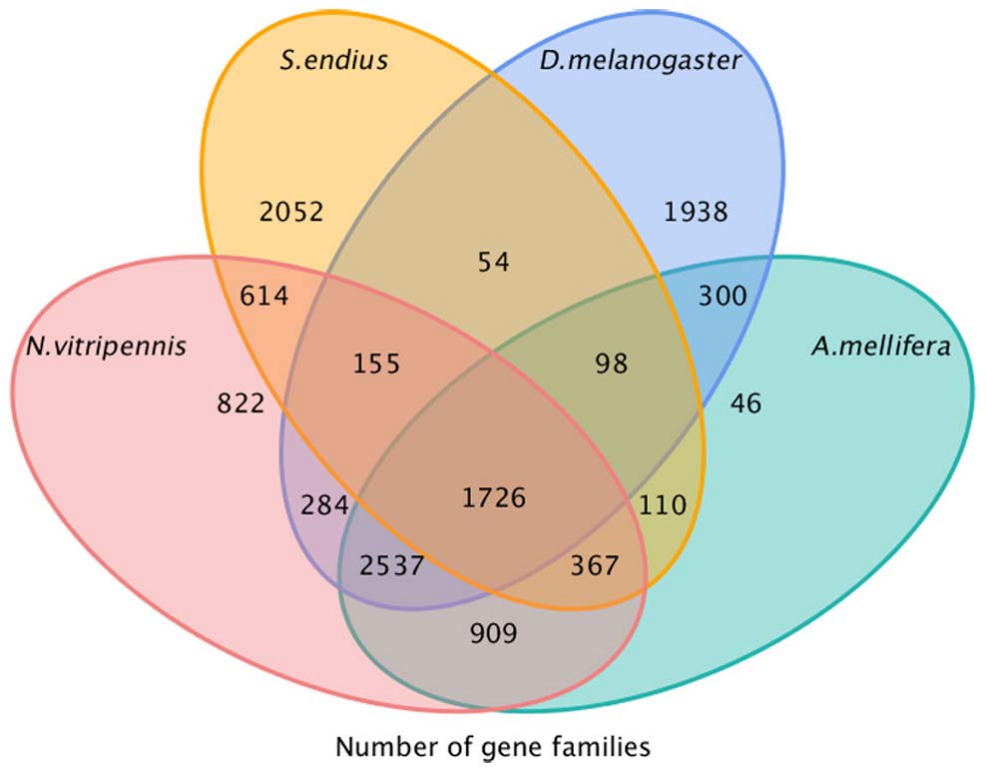
Summary of gene family classification among four species, *S. endius*, *N. vitripennis*, *A. florea* and *D. melanogaster*.

**Figure 3:**
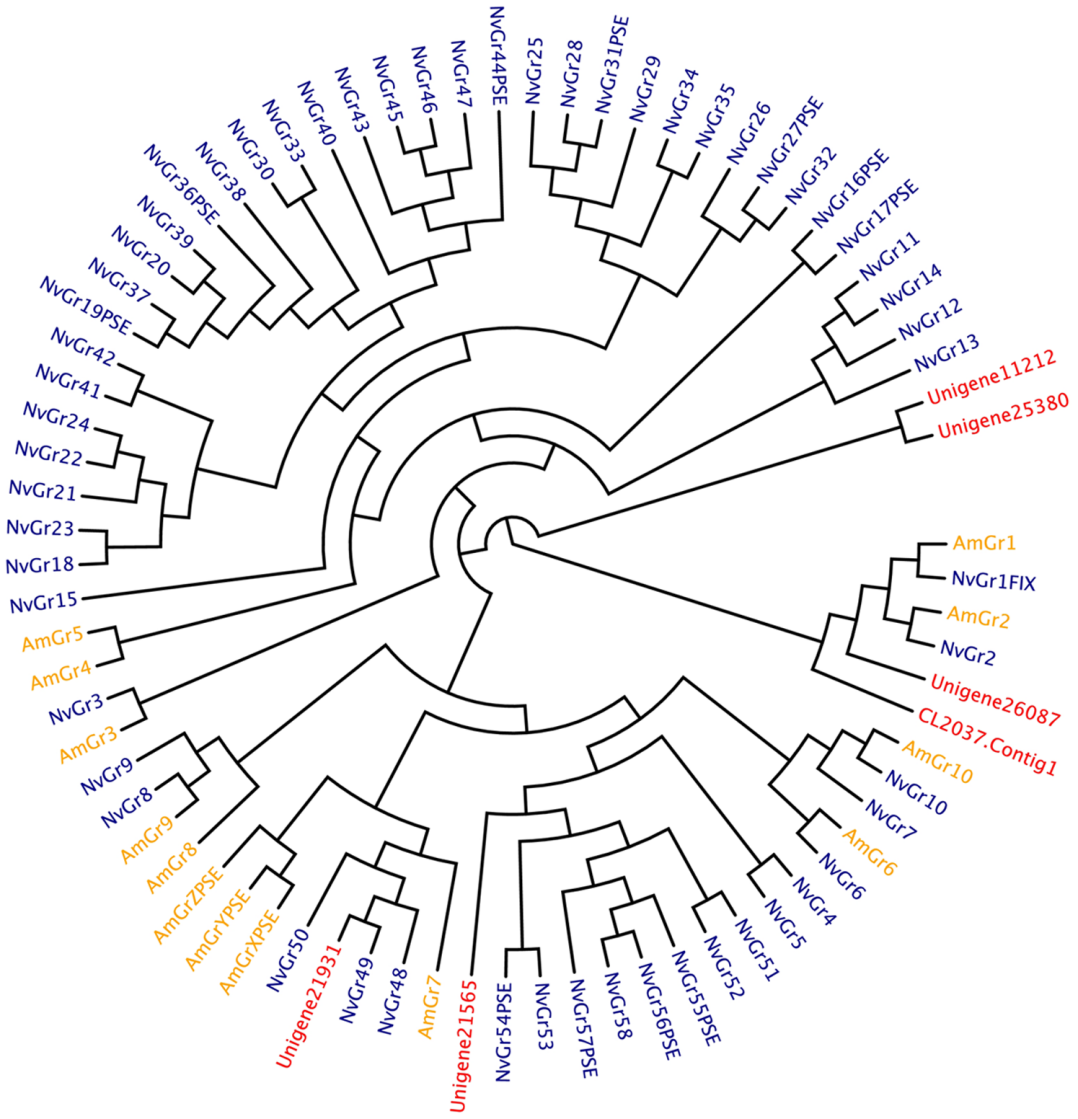
Phylogenetic tree of the gustatory receptor (Gr) family in *S. endius*, *N. vitripennis* and *A. mellifera*. AmGrs are marked in orange, NvGrs are in blue and PvGrs are red.

Using TreeFam and the pipeline described in the Methods, we obtained 3494 gene families and 567 unique gene families of *S. endius* among these four species (Figure 2). The results revealed that the majority of families (77.73%) were shared with *N. vitripennis* (also belonging to the Pteromalidae family) followed by *A. florea* (61.71%) (Hymenoptera) and *D. melanogaster* (54.78%) (Figure 3). This result corresponds to their phylogenetic positioning.

A significant percentage of transcripts (63.83%) in *S. endius* were found not to be from a conserved lineage. This could be attributed to the presence of novel families. Alternatively, the derived transcripts may be from chimeric sequences (assemblage errors) and non-conserved areas of proteins where homology is not detected, in agreement with several other transcriptomic studies [55]–[58]. The KEGG enrichment in these transcripts contained RNA transport, aminoacyl-tRNA biosynthesis, *Vibrio cholerae* infection, non-homologous end-joining, caffeine metabolism, adherens junction and proteasome (Table S3). These mainly involved transport and biosynthesis of RNA and protein. Surprisingly, *Vibrio cholerae* infection was enriched in the present study. *Vibrio cholerae* is a major cause of mammalian and human morbidity and mortality in many parts of the world [59]. Blow and colleagues [60] reported that additional virulence factors are required for intoxication of *D. melanogaster* that may not be essential for intoxication of mammals, and therefore the fly or a related arthropod may be a true host of *V. cholerae* in nature. This is in agreement with our results. The GO enrichment of these transcripts/families suggests that they are mainly involved in the processes of metabolism and protein biosynthesis (Table S4).

### Chemoreceptor Superfamily

To identify the chemoreceptor superfamily, a homologous method was performed. A total of six gustatory receptor (Gr) genes were identified in *S. endius* sequences. CL2037 contained five splice isoforms (CL2037_contig1 to CL2037_contig5), but all the transcripts have a low expression level (<1 FPKM). Only Unigene11212 has a high expression level (34.2 FPKM) from all these Gr genes, with the others having levels less than three FPKM. CL2037 and Unigene26087 are orthologs of Sugar receptors (NvGr1 and NvGr2). The phylogenetic tree of 78 Gr genes, which contained the Gr genes in *P. vindemmiae*, *N. vitripennis*, and *A. florea*, is shown in Figure 3.

**Figure 4:**
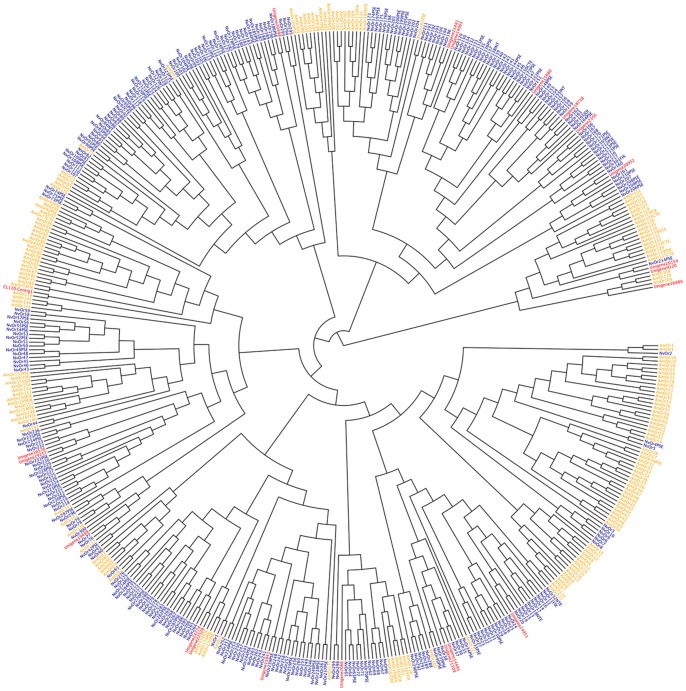
Phylogenetic tree of the odorant receptor (Or) family in *S. endius*, *N. vitripennis* and *A. mellifera*. AmGrs are marked in orange, NvGrs are in blue and PvGrs are red.

A total of 21 odorant receptor (Or) genes were identified in *S. endius* sequences. CL110 contained 12 splice isoforms (CL110_contig1 to CL110_contig12), but all the transcripts have a low expression level (<1 FPKM). Only Unigene9120 has a high expression level (11.5 FPKM) from all these Or genes, with the others having levels less than three FPKM. A total of 496 Or genes were constructed into a phylogenetic tree, and the results are shown in Figure 4.

### Putative Molecular Markers

Transcriptomes are an important resource for the rapid and cost-effective development of genetic markers [61]. The molecular markers derived from the transcribed regions are more conservative, providing a greater potential for identifying functional genes. Among the various molecular markers, simple sequence repeats (SSRs) are highly polymorphic, easier to develop, and serve as a rich resource of diversity [62]. To detect new molecular markers, all of the unigenes were scanned using the MISA Perl script (http://pgrc.ipk-gatersleben.de/misa/). In total, 3097 (8.51%) unigenes contained 3727 cDNA-SSR markers (Table S4 and Table S6). In these cDNA-SSRs, the di-nucleotide (1972) and tri-nucleotide (1285) repeat motifs had the highest frequencies, followed by the mono-nucleotide repeats (355), hexa-nucleotide repeats (54), quad-nucleotide repeats (45) and penta-nucleotide repeats (16). After designing and filtering primers, 719 cDNA-SSR markers were found to have at least one primer (Table S5 and Table S7). These data could provide a platform for better understanding of the polymorphisms of *S. endius*.

## Discussion

RNA-seq is a powerful and cost-effective strategy for obtaining many functional genes in non-model organisms. In this study, to obtain the highest quality of *S. endius* genes, we applied deep sequencing and *de novo* assembly RNA-seq. Nearly ten Gbp of data were yielded by the Hiseq platform, with more than 90% Q20 bases. A total of 36319 transcripts were acquired, with a total length and N50 length of 54072690 bp and 2956 bp, respectively (Figure 1 and Table 1). The total reads used may represent the completeness of assembly of a *de novo* transcriptome. This was considered an important factor for evaluating the completeness of assembled transcriptomes without a reference in the chili pepper and the chickpea [26], [62], where their highest ratio was 85.74% and 82.8%, respectively. In the present study, 93.15% of the reads were realigned back to unigenes using SOAP2, and the coverage of unigenes had a positive relationship when compared with the length of the given unigenes. These results suggested that we obtained the majority of transcripts in the present data. Another important factor used to assess the sequencing of the chili pepper was the accuracy of assembly. The results of our evaluation showed that 98.91% of transcripts were completely covered by at least one read, which is also an improvement on the sequencing of the chili pepper (95.5%) [26]. Combined with the N50 length, these results show that we obtained a high quality transcriptome by *de novo* assembly. This high quality may be due to our deep sequencing data (9983 megabases).

Chemoreception is important for locating food, mates, hosts and other resources in many parasitoid insects. Host location is a very important behavior for maintaining the breeding population in the parasitoid wasp. Schurmann and colleagues [63] have shown that females learn host odors and avoid unfamiliar odors in subsequent host-seeking, which may facilitate their ability to find relatively rare fly pupae in widely dispersed bird nests. The insect chemoreceptor superfamily was first identified in *D. melanogaster*, and consists of the odorant receptor (Or) family and the gustatory receptor (Gr) family of seven-transmembrane-domain proteins [64]–[68]. In this study, we identified the expression of six Gr and 21 Or genes in the present sample. Interestingly, there was one gene in each of the families with a higher expression level (Unigene11212∶34.2 FPKM in the Gr family, and Unigene9120∶11.5 FPKM in the Or family). A possible cause for this high expression of a single gene could be that the wasp was reared under laboratory conditions, with less complex surroundings than in the wild. These included only one species of host, adequate food and lack of predators or other pressure. In this situation, the wasp may reduce its gene expression to the few genes necessary for maintaining life, although it has the ability to deal well with a more complex environment in the wild. Additionally, other genes have shown several alternative splice isoforms in each family. Alternative mRNA splicing (AS) is a pivotal regulatory mechanism allowing the expansion of the genome expression potential through the generation of multiple proteins from a single gene. Several splice isoforms from one gene suggests that this gene yields a set of proteins, and could therefore perform a complex function. However, the expression levels of these genes are very low (<1 FPKM). To avoid any bias resulting from our use of unique mapped reads for the calculation of the expression level, we chose the longest transcript representing this gene to calculate the expression level. The expression levels of CL2037 and CL110 were 3.61 and 7.61 FPKM, both less than the average expression level of 15 FPKM. A possible cause is that olfactory and gustatory tissue may represent only a very minor part of the body of the wasps, from which total RNA was isolated. This contradictory result of a gene expressed at low levels yet regulated by splicing could indicate the existence of a complex regulatory mechanism.

In conclusion, this study aimed to obtain fundamental molecular knowledge of *S. endius*. It contributes a significant non-redundant set of 36319 characteristic sequences using short-read sequence data and *de novo* assembly, which will provide new insights into the biology of *S. endius*. In addition, we have identified expressed genes from the Gr and Or families, which will be the subject of advanced study in the future. A number of SSR markers were also predicted, which could facilitate the identification of polymorphisms and functional genes within wasp populations.

## Supporting Information

Table S1 **The annotation of all assembly transcripts in** ***S. endius*** **.**
(XLS)

Table S2 **The Pfam domain search of** ***S. endius*** **transcripts.**
(XLS)

Table S3 **The summary of KEGG enrichment in** ***S. endius*** **-specific sequences.**
(XLS)

Table S4 **The summary of GO enrichment in** ***S. endius*** **-specific sequences.**
(XLS)

Table S5 **The putative chemoreceptor superfamily transcripts in** ***S. endius*** **.**
(XLS)

Table S6 **cSSR information derived from all transcripts.**
(XLS)

Table S7 **cSSR primer information.**
(XLS)
